# A common microvascular endophenotype in head injuries and Alzheimer’s disease

**DOI:** 10.1002/alz.092817

**Published:** 2025-01-03

**Authors:** Shangzhou Xia, Zhen Zhao

**Affiliations:** ^1^ Zilkha Neurogenetic Institute, Keck School of Medicine, University of Southern California, Los Angeles, CA USA

## Abstract

**Background:**

Cerebrovascular injury is a common pathological feature of a spectrum of neurological disorders, including traumatic brain injury (TBI), stroke, Alzheimer’s disease (AD), and aging. Vascular manifestations among these conditions are similar indeed, including the breakdown of the blood‐brain barrier (BBB). However, whether there is a unique molecular mechanism underlying the vascular changes among these conditions remains elusive.

**Method:**

We performed single‐cell RNA (scRNA‐seq) sequencing and obtained 29,114 high‐quality cells combined from ipsilateral hemispheres of WT C57BL/6J Sham mice and mTBI mice at day 1, 3 and 7.

**Result:**

Joint clustering of all groups revealed 7 cell populations using their specific genetic markers, including vEC, vcapEC, capEC, acapEC, aEC. Next, we identified differential expression genes between Sham and day 1 or day 3 or day 7 post‐mTBI within each endothelial cell cluster.

**Conclusion:**

Based on transcriptomic analysis on cerebrovascular scRNA‐seq datasets, we identified a common molecular signature between mTBI and aging vasculature, involving a novel transmembrane protein gene *Tmem252*. *Tmem252* upregulation in brain endothelial cells may represent a shared endophenotype of microvascular injuries in different pathological conditions, and therapeutically targeted for treating BBB breakdown and vascular dysfunctions.

